# Transcriptome Profiling Identifies *Multiplexin* as a Target of SAGA Deubiquitinase Activity in Glia Required for Precise Axon Guidance During *Drosophila* Visual Development

**DOI:** 10.1534/g3.116.031310

**Published:** 2016-06-01

**Authors:** Jingqun Ma, Kaelan J. Brennan, Mitch R. D’Aloia, Pete E. Pascuzzi, Vikki M. Weake

**Affiliations:** * Department of Biochemistry, Purdue University, West Lafayette, Indiana 47907; † Purdue University Libraries, Purdue University, West Lafayette, Indiana 47907; ‡ Purdue University Center for Cancer Research, Purdue University, West Lafayette, Indiana 47907

**Keywords:** histone ubiquitination, glia, axon guidance, SAGA, SCA7

## Abstract

The Spt-Ada-Gcn5 Acetyltransferase (SAGA) complex is a transcriptional coactivator with histone acetylase and deubiquitinase activities that plays an important role in visual development and function. In *Drosophila melanogaster*, four SAGA subunits are required for the deubiquitination of monoubiquitinated histone H2B (ubH2B): Nonstop, Sgf11, E(y)2, and Ataxin 7. Mutations that disrupt SAGA deubiquitinase activity cause defects in neuronal connectivity in the developing *Drosophila* visual system. In addition, mutations in SAGA result in the human progressive visual disorder spinocerebellar ataxia type 7 (SCA7). Glial cells play a crucial role in both the neuronal connectivity defect in *nonstop* and *sgf11* flies, and in the retinal degeneration observed in SCA7 patients. Thus, we sought to identify the gene targets of SAGA deubiquitinase activity in glia in the *Drosophila* larval central nervous system. To do this, we enriched glia from wild-type, *nonstop*, and *sgf11* larval optic lobes using affinity-purification of KASH-GFP tagged nuclei, and then examined each transcriptome using RNA-seq. Our analysis showed that SAGA deubiquitinase activity is required for proper expression of 16% of actively transcribed genes in glia, especially genes involved in proteasome function, protein folding and axon guidance. We further show that the SAGA deubiquitinase-activated gene *Multiplexin* (*Mp*) is required in glia for proper photoreceptor axon targeting. Mutations in the human ortholog of *Mp*, *COL18A1*, have been identified in a family with a SCA7-like progressive visual disorder, suggesting that defects in the expression of this gene in SCA7 patients could play a role in the retinal degeneration that is unique to this ataxia.

Chromatin regulators play an important role in neuronal development through their effects on gene expression. In particular, the removal of ubiquitin from monoubiquitinated histone H2B (ubH2B) is required for proper visual development (Poeck *et al.* 2001; Weake *et al.* 2008), and defects in histone deubiquitination are associated with retinal degeneration (David *et al.* 1997). Deubiquitination of ubH2B is catalyzed by the ubiquitin protease subunit of the Spt-Ada-Gcn5 Acetyltransferase (SAGA) transcriptional coactivator complex: Nonstop (FBgn0013717) in *Drosophila melanogaster*, Ubp8 in *Saccharomyces cerevisiae*, and USP22 in humans (Zhao *et al.* 2008; Henry *et al.* 2003; Weake *et al.* 2008; Zhang *et al.* 2008). SAGA’s ubiquitin protease requires three additional proteins for activity: Sgf11 (FBgn0036804), E(y)2, and ATXN7/Ataxin 7 (Kohler *et al.* 2010; Samara *et al.* 2010; Lang *et al.* 2011). Mutations in *nonstop* and *sgf11* disrupt photoreceptor axon targeting in *Drosophila* (Weake *et al.* 2008; Poeck *et al.* 2001; Martin *et al.* 1995; Berger *et al.* 2008). In humans, polyglutamine (polyQ) expansion in ATXN7 results in SCA7, a dominant neurodegenerative disorder distinguished from other ataxias by retinopathy (David *et al.* 1997; Enevoldson *et al.* 1994). Notably, polyQ-expanded ATXN7 reduces SAGA deubiquitinase activity *in vivo*; thus, defects in ubH2B deubiquitination could induce the retinal degeneration that is unique to this ataxia (McCullough *et al.* 2012; Yang *et al.* 2015; Lan *et al.* 2015).

Although SAGA associates with and deubiquitinates ubH2B at the majority of actively transcribed genes, only a subset of these genes require ubH2B deubiquitination for expression (Weake *et al.* 2011; Bonnet *et al.* 2014). Studies in yeast and flies indicate that SAGA deubiquitinase activity is required for activation of inducible, tissue-specific genes that regulate developmental processes (Henry *et al.* 2003; Weake *et al.* 2011). However, the SAGA-regulated genes that are required for visual development, and that directly result in the visual degeneration in SCA7 patients, are unknown.

Several studies indicate that proper SAGA deubiquitinase activity is required in glia, rather than neurons, for both visual development and healthy eye function. Clonal analysis indicates that *nonstop* is required in glia for their migration to the correct region of the brain where they subsequently provide appropriate termination cues to photoreceptor axons (Poeck *et al.* 2001). In addition, expression of polyQ-expanded ATXN7 in glia is sufficient to induce neurodegeneration in a SCA7 mouse model (Custer *et al.* 2006). Thus, we sought to identify genes regulated by SAGA deubiquitinase activity in glia, with the aim of identifying genes that are required in glia for proper migration, and that could lead to visual degeneration in SCA7.

Previously, we attempted to identify SAGA deubiquitinase-regulated genes that are required for neuronal connectivity by microarray analysis of gene expression in larvae (Weake *et al.* 2008). However, this approach did not identify suitable candidate genes due to the small number of lamina glial cells in the whole larvae. Here, we characterize the SAGA deubiquitinase-dependent transcriptome of glia enriched from the central nervous system and eye-antennal imaginal disc of *Drosophila* third instar larvae, and identify *Multiplexin* (*Mp*) as a target of SAGA deubiquitinase activity that is required in glia for lamina glial organization and proper photoreceptor axon targeting.

## Materials and Methods

### Genetics

The *nonstop* and *sgf11* alleles used in this study were previously described (Weake *et al.* 2008). Flies expressing the UAS-KASH-GFP transgene, *P{w^+mC^ = UAS-GFP-Msp300KASH}attP2*, were previously described (Ma and Weake 2014). The following three genotypes were used to label glial nuclei for affinity-enrichment in wild-type, *nonstop*, or *sgf11* mutant larvae respectively: w;;P{w^+m*^=GAL4}repo, *P{w^+mC^ = UAS-GFP-Msp300KASH}attP2/TM3*, *Sb^1^* for wild type, w;;P{w^+m*^=GAL4}repo, *P{ry^+t7.2^ = PZ}not^02069^ry^506^/ P{ry^+t7.2^ = PZ}not^02069^ry^506^*, *P{w^+mC^ = UAS-GFP-Msp300KASH}attP2* for *nonstop*, and w;;P{w^+m*^=GAL4}repo, *Pbac{w^+mC^ = RB}CG13379^e01308^/PBac{w^+mC^ = RB}CG13379^e01308^*, *P{w^+mC^=[UAS-GFP-Msp300KASH}attP2* for *sgf11*. For RNAi crosses, *w;;UAS-dicer2*; P{w^+m*^=GAL4}repo}, *ro-τlacZ/TM6C*, *Tb*, *Sb* flies were crossed with the following RNAi stocks provided by the Bloomington *Drosophila* Stock Center at Indiana University (*Luciferase*: BL35788; *Mp*: BL52981, BL32921; *jing*: BL55633, BL27024; *Rab6*: BL27490, BL35744; *ras*: BL31653, BL31654; and *uzip*: BL29558) and the Vienna *Drosophila* RNAi center (*sgf11*: 17166). RNAi crosses were performed at 28° and wandering third instar larval F1 progeny were analyzed. The following two genotypes were used for analysis of *loco^rC56^* localization in wild-type or *sgf11* larvae: w;;P{ry^+*^=lacZ-un1}loco^rC56^, *PBac{w^+mC^ = RB}CG13379^e01308^* and w;;P{ry^+*^=lacZ-un1}loco^rC56^ (BL10009) (Granderath *et al.* 1999; Winberg *et al.* 1992).

### Immunohistochemistry and X-gal staining

Central nervous system/eye-antennal disc lobe complexes from wandering third instar larvae were dissected and fixed with 4% formaldehyde before immunostaining with the following antibodies: anti-chaoptin (mAb24B10, mouse, 1:10; Developmental Studies Hybridoma Bank) (Fujita *et al.* 1982); anti-repo (8D12, mouse, 1:10, Developmental Studies Hybridoma Bank) (Alfonso and Jones 2002); anti-β-galactosidase (#A11132, rabbit, 1:500, Molecular Probes); goat anti-mouse Alexa Fluor 568 (#A11004, 1:300, Life Technologies); and goat anti-rabbit Alexa Fluor 488 (#A11001, 1:300, Life Technologies). Nuclei were stained using 0.1 μg/ml 4′,6-diamidino-2-phenylindole (DAPI, #40011, Biotium). Laser scanning confocal imaging was performed using a Nikon A1R inverted confocal microscope under a 40 × /1.30 NA oil immersion Nikon Plan Fluor objective. Confocal images are presented either as single planes or as 3-D maximum projection images consisting of 0.5–1.0 μm *z*-stacks using NIS-Elements software. X-gal staining of central nervous system/eye-antennal disc complexes from wandering third instar larvae was performed as described (Sweeney *et al.* 2012) with the following modifications: dissected central nervous system/eye-antennal disc complexes were fixed in 1% formaldehyde prior to staining, and 0.3% Triton X-100 was included in the X-gal staining solution. Stained complexes were examined using a Zeiss Discovery V12 light microscope.

### Glial nuclear RNA isolation and RNA sequencing (RNA-seq)

Central nervous system/eye-antennal disc complexes were dissected from wild-type, *nonstop*, and *sgf11* wandering third instar larvae. GFP-labeled glial nuclei were enriched from 400 dissected eye-brain complexes for each biological replicate and genotyped as previously described (Ma and Weake 2014). Total nuclear RNA was extracted from isolated glial nuclei using Trizol reagent (Life Technologies), treated with DNase (Roche), and mRNA was enriched and purified using an RNeasy MinElute Cleanup Kit (#74204, QIAGEN). RNA (7 ng) was used to generate double-stranded cDNA for each sample using the Ovation RNA-Seq System V2 (#7102, NuGEN technologies). Downstream indexed TruSeq PCR-free DNA libraries (Illumina) were constructed from amplified, double-stranded cDNA. All 12 samples were added to a single pool that was clustered in two lanes of a HiSequation 2500 paired-end v3 high output flowcell to generate two 101 base reads per cluster.

### RNA-seq data analysis

Four biological replicates were analyzed for each of the following genotypes: wild type, *nonstop*, and *sgf11*. Quality trimming was performed on paired-end reads for all 12 samples using Trimmomatic (v0.32) (Bolger *et al.* 2014) to remove bases with Phred33 < 30, resulting in properly paired reads of at least 50 bases. Quality trimmed reads were mapped against the bowtie-2 (v2.2.4) (Langmead and Salzberg 2012) indexed *D. melanogaster* genome (Drosophila_melanogaster.BDGP5.78) using Tophat (v2.0.13) (Trapnell *et al.* 2009). The raw counts matrix was generated by Htseq-count (v0.6.1) applying no strand-specific assay, union mode, and default parameters (Anders and Huber 2010). Differential expression analysis was performed on genes with greater than one count per million (CPM) in at least four of the 12 samples. Differentially expressed genes were detected in each mutant genotype relative to the wild-type genotype using edgeR (Robinson *et al.* 2010) using a False Discovery Rate (FDR) of less than 0.01. The distance matrix and scatter plots were generated using Bioconductor packages of DESeq2 (Love *et al.* 2014) and edgeR (Robinson *et al.* 2010), respectively, in R (v3.1.2). Gene Ontology (GO) term enrichment analysis was performed using a Fisher’s exact test and significantly enriched GO terms were defined as those with a FDR < 0.001. Actively transcribed genes were defined as fragments per kilobase of transcript per million mapped reads (FPKM) of greater than one in wild-type glia. The GO term analysis used the Bioconductor *Drosophila* genome annotation package 3.1.2 with GO data from March 17, 2015. GO terms with less than eight or more than 250 genes were removed, as were gene annotations with no supporting data.

The RNA-seq data for the central nervous system of OregonR larvae were obtained from the modENCODE project: Dm Tissue Expression RNA-seq third instar larvae central nervous system sequences (ModENCODE_4257). Two biological replicates for larval central nervous system RNA-seq data were mapped against the bowtie-2 (v2.2.4) (Langmead and Salzberg 2012) indexed *D. melanogaster* genome (Drosophila_melanogaster.BDGP5.78) using Tophat (v2.0.13) (Trapnell *et al.* 2009). A raw counts matrix was generated by Htseq-count (v0.6.1) applying no strand-specific assay, union mode, and default parameters (Anders and Huber 2010). A count matrix for the central nervous system and the wild-type glia nuclei was assembled, and edgeR was used to normalize libraries and determine the FPKM values for genes that had greater than one CPM in two or more of the six samples.

### qRT-PCR analysis

Exonic primers flanking intron regions of target genes were designed for quantitative reverse transcription polymerase chain reaction (qRT-PCR) analysis using Primer3. qRT-PCR analysis was performed on cDNA as previously described (Ma and Weake 2014).

### Data availability

All *Drosophila* strains are available upon request. The RNA-seq expression data discussed in this publication have been deposited in NCBI’s Gene Expression Omnibus (Edgar *et al.* 2002) and are accessible through GEO series accession number GSE75681 (http://www.ncbi.nlm.nih.gov/geo/query/acc.cgi?acc=GSE75681). RNA-seq data for the central nervous system of OregonR larvae were obtained from the modENCODE project: Dm Tissue Expression RNA-seq third instar larvae central nervous system sequences (ModENCODE_4257: SRR070409 and SRR070410). FPKM values for central nervous system *vs.* glial-enriched samples are reported in Supplemental Material, Table S1. Lists of up- and downregulated genes for each mutant relative to the wild type are provided in Table S2, Table S3, Table S4, Table S5, Table S6, and Table S7. Complete edgeR analysis results are reported in Table S8 and Table S9. Primer sequences are listed in Table S10. Raw counts for RNA-seq data are provided in Table S11. All R Code used in this study is available upon request.

## Results

### Enrichment of glia from wild-type, nonstop, and sgf11 optic lobes for transcriptome profiling

The compound eye of *Drosophila* is composed of ∼800 ommatidia, each of which contain eight different photoreceptor neurons (R1–R8 cells) arranged in a stereotypical pattern. During the third larval instar, R1–R8 photoreceptors extend axons from the eye imaginal disc through the optic stalk where they project into different synaptic layers in the optic lobe: R1–R6 project into the lamina between two layers of glial cells, the epithelial and marginal glia, while R7 and R8 extend further into the medulla (Clandinin and Zipursky 2002). Mutations in *nonstop* and *sgf11* result in a failure of glial cells to migrate into the lamina and misprojection of R1–R6 axons into the medulla (Poeck *et al.* 2001; Weake *et al.* 2008). Since clonal analysis indicates that the SAGA deubiquitinase Nonstop is required in glia to regulate cell migration (Poeck *et al.* 2001), we hypothesized that SAGA deubiquitinase activity is required in glia for the expression of specific genes that regulate glial migration, which in turn controls proper photoreceptor axon targeting. However, we note the formal possibility that SAGA could also be required in glia to provide the termination signal necessary for proper axon targeting; this alternative hypothesis will be addressed later in this study.

**Figure 1:**
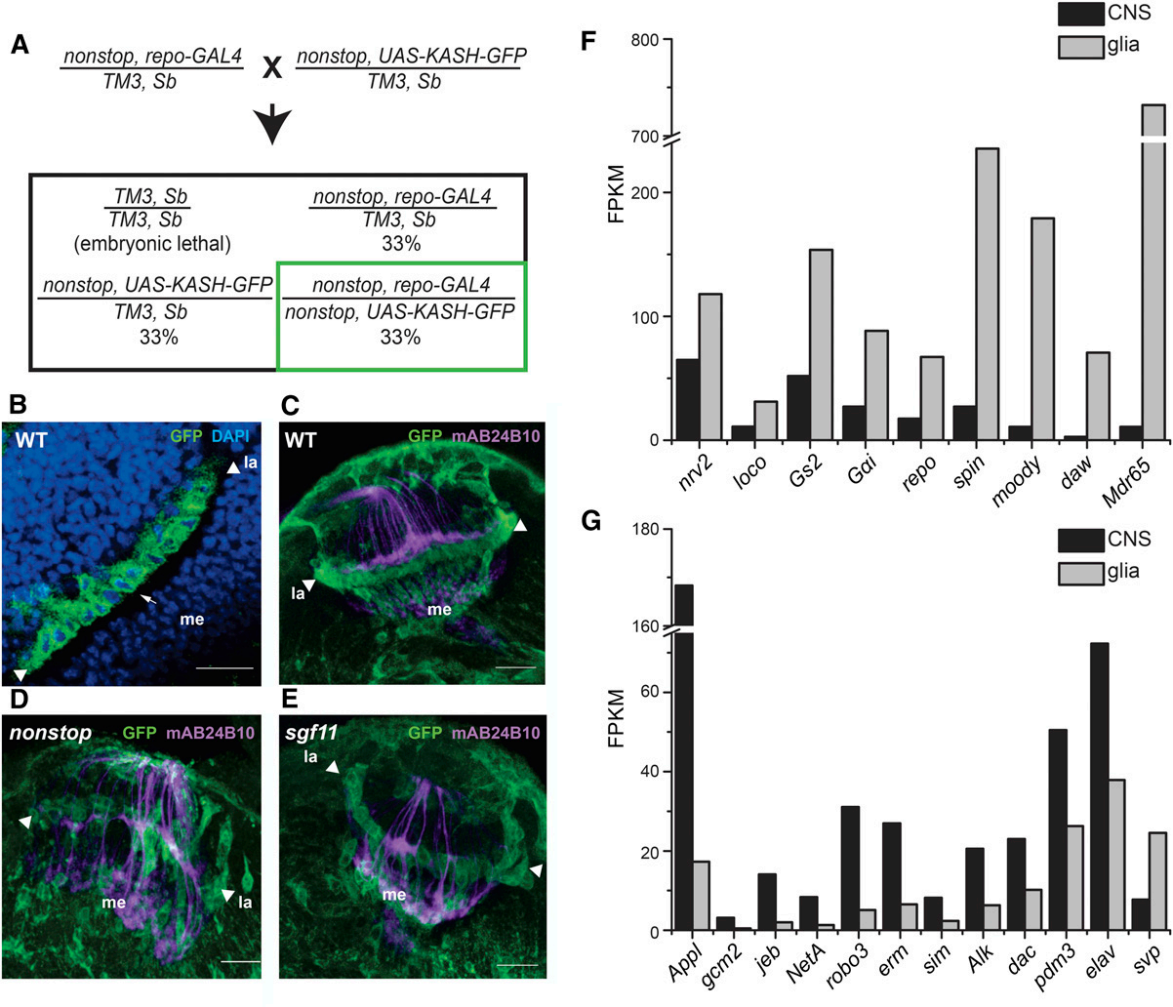
Glial nuclei are labeled with KASH-GFP in wild-type, *nonstop* and *sgf11* optic lobes. (A) Genetic scheme for positive labeling of glial nuclei in *nonstop* or *sgf11* homozygous mutant larvae. The expected percentage of progeny with each genotype at the larval stage is indicated. (B) Glial nuclei in a wild-type optic lobe were positively labeled with KASH-EGFP (GFP, green) and stained with DAPI (blue). Arrowheads mark the edges of the lamina (*la*), and the medulla (*me*) is indicated. A single KASH-GFP labeled glial nuclei is indicated by the arrow. Scale bars, 20 μm. (C–E) Glial nuclei in wild-type (C), *nonstop* (D), and *sgf11* (E) optic lobes were positively labeled with KASH-EGFP (GFP, green). Mutant genotypes (panels D and E) were labeled using the genetic approach described in panel A. In third instar larvae, R1–R8 photoreceptor axons (anti-chaoptin, mAB24B10, magenta) extend axons from the eye imaginal disc into the optic lobe where these terminate in either the lamina (R1–R6, *la*) or medulla (R7–R8, *me*). In the wild-type optic lobe, R1–R6 photoreceptor axons form a densely packed growth cone, whereas in *nonstop* and *sgf11* optic lobes, a subset of photoreceptor axons project through the lamina into the medulla. Arrowheads mark the edge of lamina. Scale bars, 20 μm. (F) Bar graph indicating the transcript level (FPKM) for glial markers in affinity-enriched glia (glia: this study) relative to the larval central nervous system (CNS: ModEncode). (G) Bar graph indicating the transcript level (FPKM) for neuronal markers in affinity-enriched glia relative to the larval central nervous system. CNS, central nervous system; DAPI, 4′,6-diamidino-2-phenylindole; EGFP, enhanced green fluorescent protein; FPKM, fragments per kilobase of transcript per million mapped reads; GFP, green fluorescent protein; KASH, Klarsicht, Anc-1, and Syn3-1 homology; WT, wild-type.

As a first step in identifying novel SAGA-regulated genes that are required in glia for neuronal targeting, we sought to identify genes regulated by SAGA deubiquitinase activity, specifically in optic lobe glia. To do this, we utilized our previously developed nuclei affinity purification protocol to enrich glial nuclei from the eye imaginal disc and optic lobes of wild-type, *nonstop*, and *sgf11* third instar larvae for transcriptome profiling (Ma and Weake 2014). This protocol utilizes antibodies coupled to magnetic beads to affinity enrich nuclei labeled with GFP fused to the Klarsicht, Anc-1, and Syn3-1 homology (KASH) domain of Msp300, which localizes EGFP to the cytoplasmic face of the nuclear membrane and is expressed under Gal4/UAS control (*UAS-KASH-GFP*) (Yu *et al.* 2006; Patterson *et al.* 2004; Fischer *et al.* 2004). To label glial nuclei with KASH-GFP, we crossed flies carrying the *UAS-KASH-GFP* transgene with the glial-specific *repo-Gal4* driver (Xiong *et al.* 1994; Halter *et al.* 1995; Sepp *et al.* 2001). To label glial nuclei in *nonstop* and *sgf11* mutant larvae with KASH-GFP, we crossed flies carrying the *nonstop* or *sgf11* mutant allele on the same chromosome as *repo-GAL4* to flies with the mutant allele on the same chromosome as *UAS-KASH-GFP*, as described by the genetic scheme outlined in Figure 1A. Using this approach, only glial nuclei in homozygous *nonstop* or *sgf11* mutant larvae are labeled with KASH-GFP. We do not obtain homozygous *nonstop* or *sgf11* adult flies since these mutations result in late larval/early pupal lethality.

To test if glial nuclei were successfully labeled with KASH-GFP in each genotype, we examined the optic lobes dissected from third instar larvae using confocal microscopy. We immunostained the dissected eye-brain complexes with anti-chaoptin, which labels R1–R8 photoreceptor axons, to examine photoreceptor projections for comparison in each genotype. In optic lobes dissected from third instar larvae expressing *UAS-KASH-GFP* under *repo-Gal4* control, we observe GFP surrounding a DAPI-positive region in glial cells, consistent with nuclear envelope localization of the KASH-GFP tag (Figure 1B, arrow). Notably, we only observe GFP-labeled glia in a subset of the larval progeny from the *nonstop* or *sgf11* crosses, as expected from the genetic scheme described in Figure 1A. Further, progeny from the *nonstop* or *sgf11* crosses that exhibit GFP-labeled glia also show defects in photoreceptor axon targeting when compared with the wild type (Figure 1, C–E). Whereas in wild-type optic lobes, photoreceptor axons form thick growth cones in the lamina plexus (Figure 1C), in *nonstop* and *sgf11* optic lobes, many photoreceptor axons project through the lamina and terminate improperly in the medulla (Figure 1, D–E). Thus, glial nuclei were positively labeled with KASH-GFP in optic lobes from each of the wild-type, *nonstop*, and *sgf11* genotypes, enabling us to subsequently enrich these labeled nuclei using affinity purification (Ma and Weake 2014).

### Enriched glial nuclei express higher levels of glial-specific gene markers relative to the whole central nervous system

Since glial cells constitute no more than 10% of the total cells present in the central nervous system (Edwards and Meinertzhagen 2010), we sought to determine the level of enrichment of glial-expressed genes relative to the entire larval central nervous system in our affinity enriched nuclei. Initially, we examined levels of GFP transcripts in samples pre- and postaffinity enrichment, and found that GFP levels were 10–30 fold higher in glial nuclei from wild-type larvae following affinity enrichment (Figure S1), indicating that our samples are enriched for the GFP-labeled nuclei population of interest.

To estimate the enrichment of RNAs for glial-specific genes in our isolated nuclei, we compared gene expression in our wild-type glial samples to gene expression for the larval central nervous system. The libraries were normalized and gene expression (FPKM) was estimated with edgeR (Table S1). The expression level of genes that have previously been shown to be preferentially expressed in either glia or neurons was examined. The glial marker *repo* is expressed at ∼fourfold higher levels in the wild-type glia samples compared to the central nervous system (Figure 1F). However, four other glial-specific genes, *spin*, *moody*, *dawdle (daw)*, and *Multi drug resistance 65 (Mdr65)*, show much higher enrichment ranging from 9–68-fold (Bainton *et al.* 2005; Mayer *et al.* 2009; Zhu *et al.* 2008; Yuva-Aydemir *et al.* 2011). In contrast, several other glial marker genes including *nervana 2* (*nrv2)*, *locomotion defects (loco)*, *Glutamine synthetase 2* (*Gs2)*, and *G protein* α *i subunit* *(G*α*i)* are enriched between two- and threefold (Thomas and van Meyel 2007; Granderath *et al.* 1999; Freeman *et al.* 2003; Pereanu *et al.* 2005; DeSalvo *et al.* 2014). We note that the larval central nervous system RNA-seq data used for this comparison represents polyadenylated mRNA isolated from bulk tissue. Since our approach examines nuclear RNA rather than total cellular RNA, it is possible that the different levels of enrichment observed for these glial markers could represent temporal differences in transcription relative to total cellular mRNA levels. Other studies that have compared active transcription with steady-state mRNA by identifying intron regions (iRNA-seq) or nascent transcripts (GRO-seq) have shown that there is a lag of minutes to a few hours between acute changes in transcription and detectable changes in steady-state mRNA levels (Madsen *et al.* 2015; Step *et al.* 2014).

Next, we examined the expression level of several genes that have been shown to be expressed in neurons or their precursors. The well-characterized neuronal marker *elav* shows a twofold reduction in expression levels in the enriched-glial samples relative to the whole central nervous system (Figure 1G) (Robinow and White 1991). In addition, the transcription factor *gcm2*, which is expressed in glial and neuronal progenitor cells, shows an eightfold reduction in expression (Chotard *et al.* 2005). Other neuronal markers such as β *amyloid protein precursor-like* (*Appl*), *jelly belly (jeb)*, *Netrin-A (NetA)*, *roundabout 3 (robo3)*, *earmuff (erm)*, *single-minded (sim)*, and *Anaplastic lymphoma kinase (Alk)* show a 3–10-fold reduction in expression levels relative to the central nervous system (Martin-Morris and White 1990; Pecot *et al.* 2014; Bazigou *et al.* 2007; Timofeev *et al.* 2012; Pappu *et al.* 2011; Umetsu *et al.* 2006; Weng *et al.* 2010). In addition, the lamina neuron markers *dachshund (dac)* and *pou domain motif 3 (pdm3)* are reduced by twofold in the enriched-glial samples relative to the whole central nervous system (Mardon *et al.* 1994; Huang and Kunes 1996; Chen *et al.* 2012). We note that a recent study has shown that *pdm3* is also expressed in perineurial glial cells in the eye imaginal disc, possibly accounting for the relatively high expression of this gene in the glial-enriched data (Bauke *et al.* 2015). Surprisingly, we observed an enrichment rather than reduction in expression of the neuroblast marker gene, *seven up (svp)*, in the glial-enriched samples relative to the central nervous system (Chang *et al.* 2003). However, there is data supporting expression of *svp* in a subset of glial cells in embryos, suggesting that this gene might also be expressed in glial cells in the central nervous system (Beckervordersandforth *et al.* 2008).

Based on the direct comparison of GFP expression in our glial-enriched sample to the bulk nuclei prior to affinity enrichment (preisolation sample, Figure S1), we conclude that we have enriched the proportion of glial RNAs ∼10-fold relative to the entire central nervous system. Comparison of the glial-enriched data with publically available RNA-seq data for the larval central nervous system supports an enrichment of glial-specific RNAs and a reduction in the level of contaminating neuronal RNAs. However, since there are major differences in these data sets, in particular with regard to total cellular RNA *vs.* nuclear RNA, this comparison only provides a rough estimate of the level of enrichment of glial RNAs in our samples.

### Transcriptome profiling reveals genes that are coregulated by Nonstop and Sgf11 in glia

**Figure 2:**
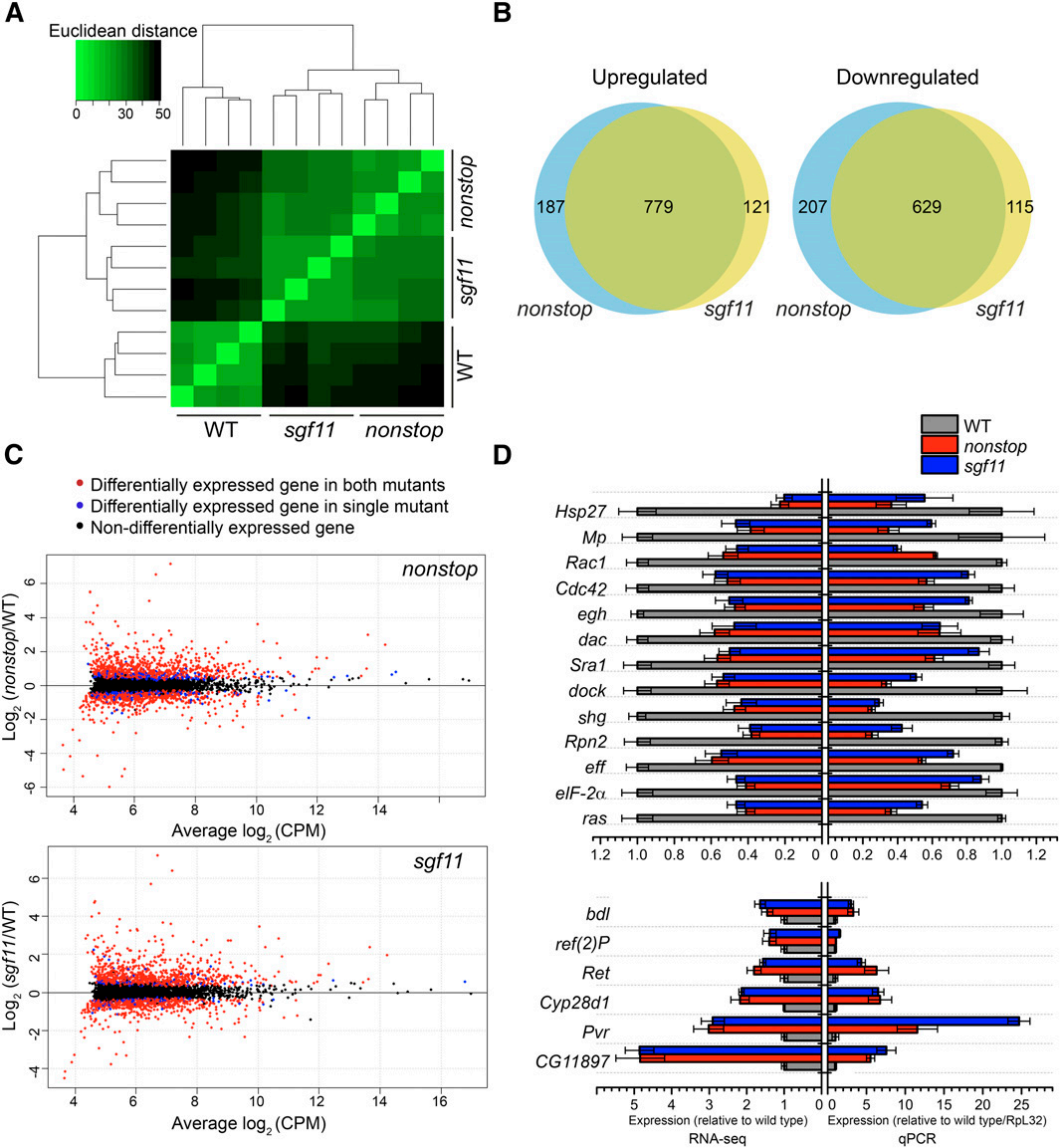
Mutations that disrupt SAGA deubiquitinase activity result in misregulated gene expression in optic lobe glia. (A) Hierarchical clustered heat map representing the Euclidean distance matrix of each genotype and biological replicate (*n* = 4) for RNA-seq analysis calculated based on regularized log_2_ transformed counts of each gene. More closely related samples are shown in light green (smallest distance, 0), and more distantly related samples are shown in black (largest distance, 50). (B) Venn diagrams indicating the number of overlapping genes that are significantly upregulated or downregulated (FDR < 0.01) in *nonstop* and *sgf11* optic lobe glia compared to wild-type optic lobe glia. (C) Scatter plots illustrating the average differential expression of each gene (dot) in mutant/wild-type glia relative to its average expression across all samples. Each gene is plotted based on the log_2_ expression ratio in either the *nonstop* (upper panel) or *sgf11* (lower panel) mutant/wild type (*y*-axis) relative to the log_2_ of its average expression level in CPM across all genotypes (*x*-axis). Genes that were identified as being significantly differentially regulated in both mutant genotypes are shown in red, and genes identified as significantly differentially regulated only in a single mutant genotype are shown in blue. (D) qRT-PCR analysis of transcript levels in cDNA from wild-type, *nonstop*, and *sgf11* optic lobe glia for a subset of the differentially regulated genes was compared with fold changes observed in the RNA-seq analysis. Mean transcript levels for each gene were normalized to *RpL32* and plotted relative to the wild type, which was set to one (right panel). RNA-seq result with fold change of genes of interest were plotted using raw abundance values compared to the wild type, which was set to one (left panel). Error bars denote standard error of the mean for three biological replicates for qRT-PCR analysis and four biological replicates for RNA-seq. CPM, count per million; FDR, false discovery rate; qPCR, quantitative polymerase chain reaction; RNA-seq, RNA sequencing; SAGA, Spt-Ada-Gcn5 acetyltransferase; WT, wild-type.

To identify genes that are transcriptionally regulated by SAGA deubiquitinase activity in glia, we isolated RNA from the enriched glial nuclei in *nonstop* and *sgf11* optic lobes and compared these transcriptomes with that of the wild type using RNA-seq. Four biological replicates were conducted for each genotype. We calculated the Euclidean distance matrix based on log transformed count data. This analysis showed that the transcriptome profiling of *nonstop* and *sgf11* optic lobe glia were similar to each other, and distinct from that of the wild-type glia (Figure 2A). The similarity of the *nonstop* and *sgf11* expression profiles suggests that loss of Nonstop or Sgf11 has a similar effect on gene expression, consistent with the findings of previous microarray studies and with their joint function in SAGA-mediated ubH2B deubiquitination (Weake *et al.* 2008).

To identify genes that are transcriptionally regulated by SAGA deubiquitinase activity, we therefore sought to identify genes that are coregulated by Nonstop and Sgf11. To do this, we conducted edgeR analysis to identify genes that are misregulated in *nonstop* or *sgf11* glia using a FDR of < 0.01 (Robinson *et al.* 2010). Using this approach, we identified 966 (Table S2) and 836 (Table S3) genes as significantly up- or downregulated, respectively, in the *nonstop* glia relative to wild-type glia, and 900 (Table S4) and 744 (Table S5) genes as significantly up- or downregulated, respectively, in the *sgf11* glia relative to wild-type glia (Figure 2B). Consistent with the similarity of the *nonstop* and *sgf11* expression profiles (Figure 2A), the majority of differentially expressed genes identified in *nonstop* and *sgf11* glia were overlapping. We identified 779 (Table S6) genes as being significantly upregulated in both *nonstop* and *sgf11* glia as compared to wild-type glia, and 629 (Table S7) genes as being significantly downregulated in both *nonstop* and *sgf11* glia (Figure 2B). Further, when we examined the change in transcript level of the significantly up- or downregulated genes in each mutant genotype relative to the wild type, and plotted this against the expression level of each gene, we observed that most of the differentially expressed genes that were identified in only one mutant exhibited smaller fold changes relative to those genes that were coregulated by Nonstop and Sgf11 (Figure 2C). Hence, we chose to focus our further analysis on the commonly up-and downregulated genes identified in *nonstop* and *sgf11* glia relative to wild-type glia. These Nonstop and Sgf11 coregulated genes will be hereafter referred to as SAGA deubiquitinase-regulated genes.

To validate the transcript level changes observed in our RNA-seq analysis, we analyzed transcript levels of 13 significantly downregulated genes and six significantly upregulated genes of potential biological relevance in wild-type, *nonstop*, and *sgf11* glia by qRT-PCR. The transcript level of each gene was normalized to the ribosomal gene *RpL32*, which is not transcriptionally regulated by SAGA (Weake *et al.* 2011). As expected from the RNA-seq analysis, all 13 of the significantly downregulated genes examined showed lower transcript levels in *nonstop* and *sgf11* glia relative to the wild type (upper panel, Figure 2D). Moreover, with the exception of *ref(2)P*, the other five upregulated genes examined showed increased transcript levels in *nonstop* and *sgf11* glia relative to wild-type glia. Therefore, we conclude that 1408 genes are regulated either directly, or indirectly, by SAGA deubiquitinase activity in glia, corresponding to ∼16% of actively transcribed genes in these glia. This number of SAGA deubiquitinase-regulated genes in glia corresponds well with previous studies in embryonic muscle that identified ∼14% of SAGA-bound, actively transcribed genes as being regulated by SAGA deubiquitinase activity (Weake *et al.* 2011).

### SAGA deubiquitinase activates expression of genes involved in proteasomal degradation, protein folding, and axon guidance

**Figure 3:**
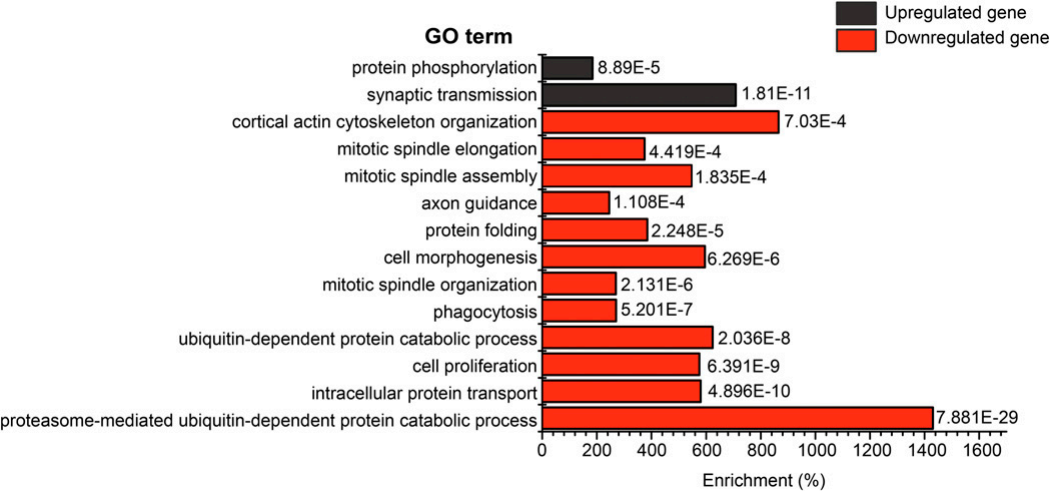
SAGA deubiquitinase-regulated genes are enriched for Gene Ontology terms including proteasome function, protein folding, and axon targeting. Significantly enriched biological process GO terms (FDR < 0.001) of upregulated genes (black) and downregulated genes (red) in both *nonstop* and *sgf11* optic lobe glia. Adjusted *p*-values for each GO term are shown to the right of each bar. Enrichment (x-axis) represents the fold increase of the number of genes in each GO term over the number expected by chance. FDR, false discovery rate; GO, Gene Ontology; SAGA, Spt-Ada-Gcn5 acetyltransferase.

To elucidate the characteristics of the SAGA deubiquitinase-regulated genes in glia, we examined the 1408 SAGA deubiquitinase-regulated genes for enrichment of specific GO terms. To do this, we conducted overrepresentation analysis relative to the entire genome separately for the upregulated and downregulated gene lists using Fisher’s exact test with a FDR of 0.001. Using this approach, we identified two and 12 biological processes as being significantly enriched in SAGA deubiquitinase up- and downregulated genes, respectively (Figure 3). Intriguingly, SAGA deubiquitinase activity appears to be important for full expression of genes involved in the proteasome-mediated protein catabolic process. Strikingly, 29 out of the 50 genes annotated as proteasome subunits, including *Regulatory particle non-ATPase 2* (*Rpn2*) (Figure 2D), are downregulated in SAGA deubiquitinase mutant glia. In addition to protein degradation, SAGA deubiquitinase activity also positively regulates the expression of genes involved in protein folding. One of the most highly downregulated genes identified in SAGA deubiquitinase mutants relative to the wild type, *Heat shock protein 27* (*Hsp27*), encodes a molecular chaperone required for proper protein folding (Figure 2D). In addition to these processes that play a critical role in glial function, we identified genes involved in axon guidance as being significantly enriched among the SAGA deubiquitinase downregulated genes.

### The SAGA deubiquitinase target Mp is required in glia for proper axon targeting

**Figure 4:**
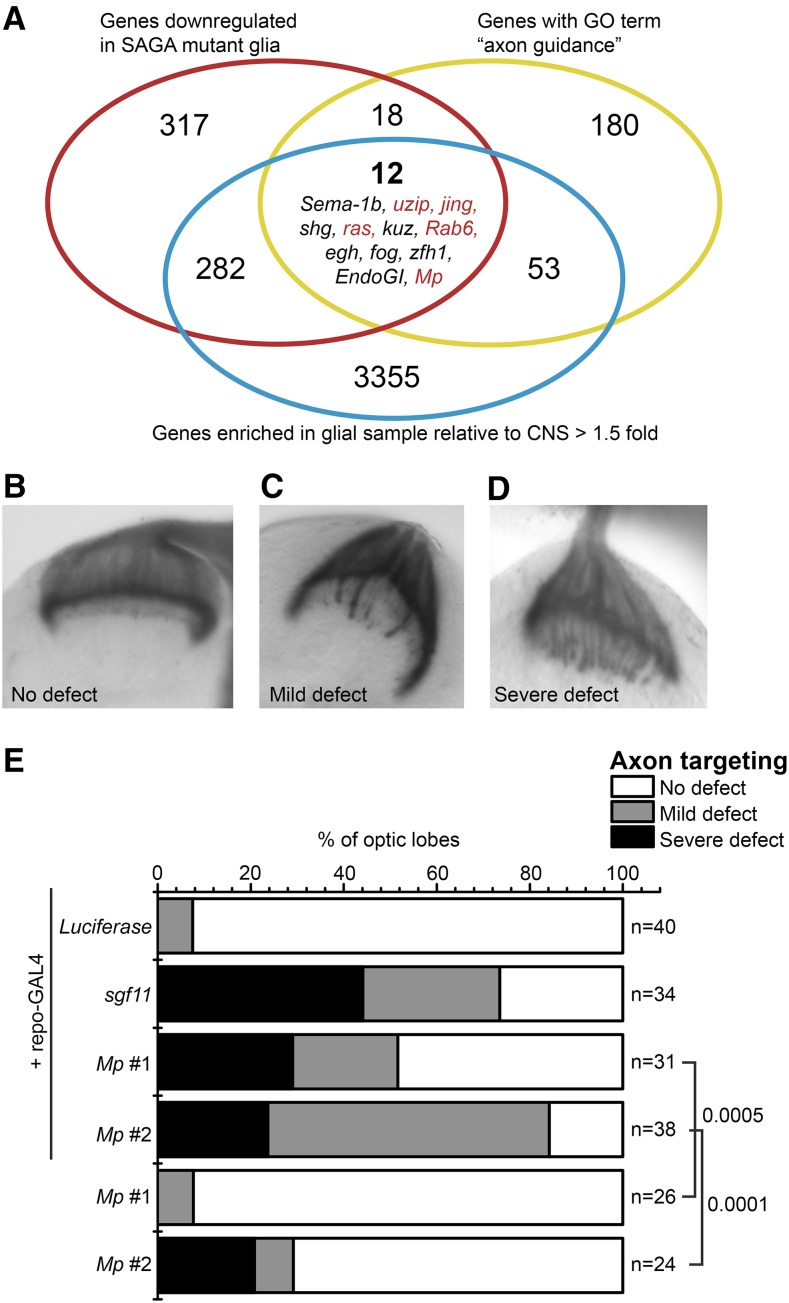
SAGA deubiquitinase-regulated gene *Multiplexin* is required in glia for proper photoreceptor axon targeting. (A) Venn diagram showing the overlap between genes expressed in glia greater than one-and-a-half-fold increase relative to the central nervous system, genes that are significantly downregulated in both *nonstop* and *sgf11* glial relative to the wild type (FDR < 0.01), and genes with GO terms that contain the term axon guidance. Genes selected for RNAi screening are highlighted in red. (B–D) Axon targeting defects were examined using X-gal staining of the R2–R5 axon marker *ro*-τ*lacZ* and classified as either no defect (mistargeted axons ≤ 1, panel B), mild defect (mistargeted axons ≤ 4, panel C), or severe defect (mistargeted axons ≥ 5, panel D). Representative images for each category from glial-specific expression of RNAi against *Luciferase* (panel B), *Mp* (panel C), and *sgf11* (panel D) are shown. (E) Stacked bar graph indicating the percentage of optic lobes exhibiting defects in R1–R6 photoreceptor axon targeting upon expression of RNAi against the indicated genes expressed in glia under *repo-GAL4* control. n, number of optic lobes analyzed. Fisher’s exact test was performed between each *Mp* RNAi genotype (two independent lines) ± *repo-Gal4* and the *p*-value is indicated for each comparison next to each bar. CNS, central nervous system; FDR, false discovery rate; GO, Gene Ontology; RNAi, RNA interference; SAGA, Spt-Ada-Gcn5 acetyltransferase.

To test the hypothesis that SAGA deubiquitinase activity regulates the expression of genes that are required in glia for proper photoreceptor axon targeting, we sought to identify SAGA-regulated genes that were expressed in glia with potential functions in controlling axon guidance. To do this, we compared the list of 629 genes that were significantly downregulated in SAGA deubiquitinase mutant glia (Table S7) with the 3701 genes that were expressed in glia (FPKM glial/CNS > 1.5), and the 263 *Drosophila* genes with GO term annotations that included the term axon guidance. Based on these criteria, we selected 12 genes that were present in all three groups for further analysis (Figure 4A). To test if these genes were required in glia for proper photoreceptor axon targeting, we examined photoreceptor projections following glial-specific expression of two independent RNAi constructs against four of these genes: *jing* (FBgn0086655), *raspberry* (FBgn0003204), *Rab6* (FBgn0015797), and *Multiplexin* (FBgn0260660). We also tested the one available RNAi line against a fifth gene, *unzipped* (FBgn0004055). The remaining genes were not examined in this study because we could either not obtain RNAi-expressing lines for these genes, or they were not as highly expressed in glia relative to the central nervous system as compared with the other five genes. We expressed RNAi constructs in glia using the *repo-Gal4* driver and examined photoreceptor axon targeting using the R2–R5-specific marker *ro*-τ*lacZ* (Garrity *et al.* 1996; Sepp *et al.* 2001). Axon targeting was examined for each RNAi line in the presence and absence of the Gal4 driver to control for nonspecific expression of RNAi. Axon targeting defects were classified based on the number of misprojected axons as either no defect (mistargeted axons ≤ 1; Figure 4B), mild defect (mistargeted axons ≤ 4; Figure 4C), or severe defect (mistargeted axons ≥ 5; Figure 4D*)*. As controls, we examined photoreceptor axon targeting upon expression of RNAi against *sgf11* and the nonspecific gene, *Luciferase*. Glial-specific expression of RNAi against *sgf11* results in mild to severe axon targeting defects in 74% of optic lobes analyzed (Figure 4E). In contrast, knockdown of *Luciferase* resulted in only 3 out of 40 analyzed optic lobes showing a mild axon targeting defect (Figure 4E). Out of the five genes we examined, only one gene showed a significant defect in photoreceptor axon targeting upon glial-specific expression of RNAi relative to the minus Gal4 control for both independent RNAi lines: *Multiplexin* (*Mp*). Glial-specific expression of two independent RNAi constructs against *Mp* results in a mild to severe axon targeting defect in 52% and 84%, respectively, of the optic lobes analyzed, similar to the level of axon targeting defect observed upon expression of RNAi against *sgf11* (Figure 4E). *Mp* had previously been shown to be required in neurons for proper motor axon targeting (Meyer and Moussian 2009). Our results indicate that *Mp* is also required noncell-autonomously in glia for proper neuronal targeting. Although we were only able to identify a requirement for *Mp* in glia for photoreceptor axon targeting in the genes analyzed in this study using our strict criteria, we note that one of the other genes we examined, *jing*, showed an extremely severe axon targeting defect for one (but not both) RNAi lines tested.

### Glial-specific knockdown of Mp modestly disrupts lamina glial organization, which correlates with mistargeting of photoreceptor axons

**Figure 5:**
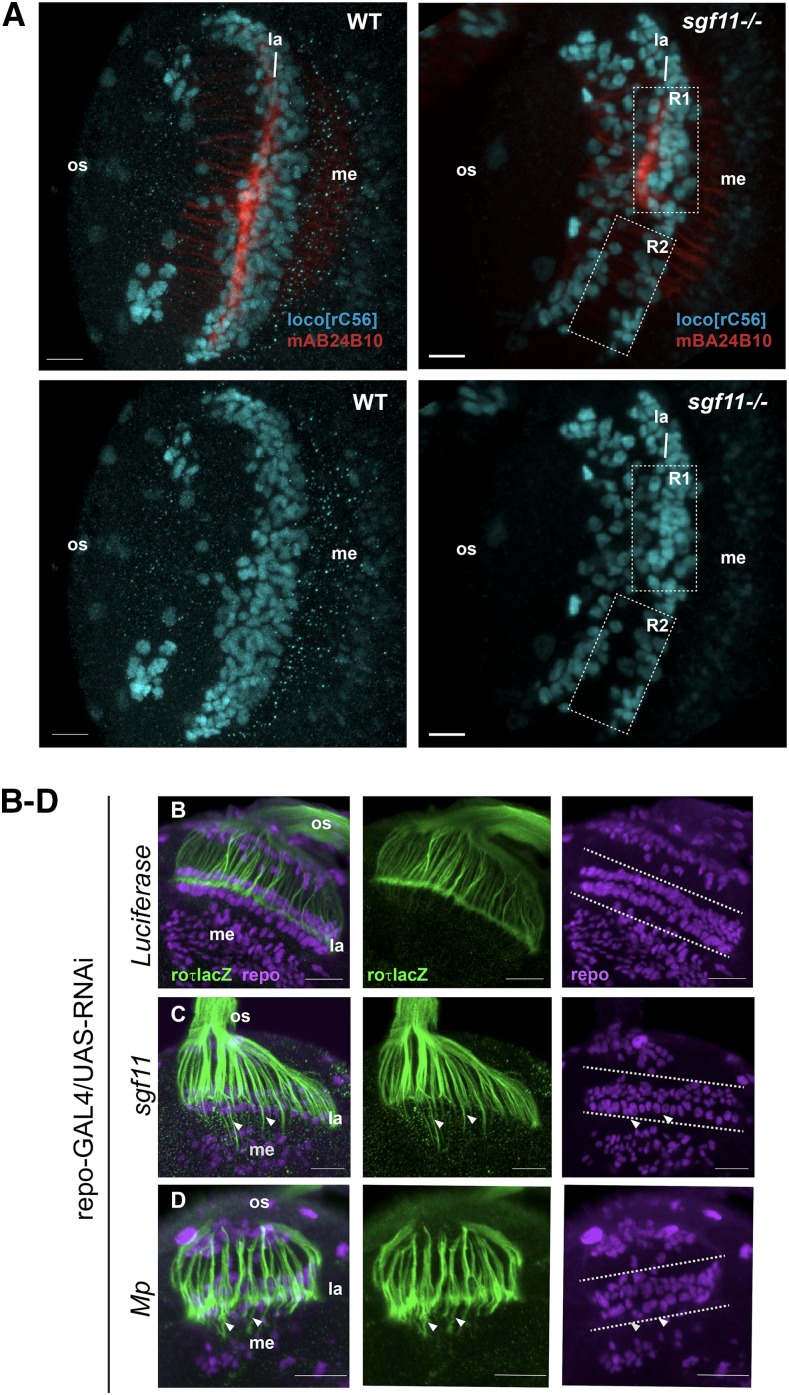
Glial-specific expression of RNAi against *Multiplexin* modestly disrupts lamina glial organization, which correlates with photoreceptor axon mistargeting. (A) Lamina glia were visualized in WT and *sgf11* optic lobes using the *loco^rC56^* marker (blue), and R1–R8 photoreceptor axons were labeled using anti-chaoptin (mAB24B10; red). In WT larvae, glia migrate appropriately to the lamina ganglia where they express the *loco^rC56^* marker. In *sgf11* larvae, although some glia migrate appropriately and express *loco^rC56^* (boxed region R1), many glia are absent from the lamina (boxed region R2), correlating with mistargeting of photoreceptor axons. Merged images for glia and axons are shown in the upper panel, and single channel images for glia alone (*loco^rC56^*) are shown in the lower panel. Optic stalk, os; Lamina, la; Medulla, me. Scale bars, 20 μm. (B–D) RNAi constructs against the indicated genes were expressed specifically in glia using the *repo-GAL4* driver, and targeting of photoreceptor axons was examined using the R2–R5 photoreceptor axon marker *ro*-τ*lacZ* (left and middle panels, green). Glial cells were labeled for comparison using anti-repo (middle and right panels, magenta). Maximum projection images of 0.5 μm z-stacks are shown for each knock-down. The positions of individual mistargeted photoreceptor axons are indicated by arrowheads in each panel. The expected position of lamina glial cells is indicated by dotted lines in the right panels for each genotype. Scale bars, 20 μm. RNAi, RNA interference; WT, wild-type.

The prevailing model for how photoreceptor axons R1–R6 find their targets in the brain is that lamina glial cells provide the signals that are necessary to control photoreceptor axon termination (Poeck *et al.* 2001). Since both *sgf11* and *nonstop* mutant larvae have strong defects in glial migration (Weake *et al.* 2008; Poeck *et al.* 2001), the simplest model for how SAGA functions in this process is that SAGA deubiquitinase activity is required for the expression of genes in glia that are necessary for their proper migration. However, one alternative possibility is that SAGA deubiquitinase-activated genes are also required in glia following migration to provide the appropriate termination signal to photoreceptor axons. While this second possibility is unlikely, neither we nor Poeck *et al.* (2001) were previously able to distinguish whether SAGA was also required in glia, following migration, to provide the termination cue necessary for axon targeting. Thus, to address this second possibility, we reexamined glial organization in *sgf11* homozygous mutant larvae using a *lacZ* enhancer trap in the *locomotion defects* locus (l*oco^rC56^*) that marks lamina glia, including both marginal and epithelial glial layers, medullary glial cells, and subretinal cells (Granderath *et al.* 1999; Winberg *et al.* 1992). In the wild type, a thick layer of lamina glial cells is observed in the lamina region (*la*) where R1–R6 photoreceptor axons terminate, forming the lamina plexus (Figure 5A). In contrast, significant numbers of these glia are absent from the lamina region in the *sgf11* mutant, concomitant with misprojection of photoreceptor axons into the medulla (Figure 5A, boxed region R2). However, as shown in the *sgf11* optic lobe in Figure 5A, in some mutant animals a subset of glia migrate appropriately to the lamina (Figure 5A, boxed region R1). Notably, in these regions of the lamina where glia are present, we observe normal photoreceptor axon termination. From this, we conclude that although SAGA deubiquitinase activity is required for glia to migrate, it is not subsequently required in glia to provide the signal necessary for proper axon termination. Based on these observations, we hypothesized that the photoreceptor axon targeting defects observed upon glial-specific expression of RNAi against the SAGA deubiquitinase-activated gene, *Mp*, would correlate with defects in lamina glial migration. Thus, we examined both glial organization and axon targeting in optic lobes in which RNAi against *Mp* had been expressed specifically in glia. To visualize photoreceptor axons, we used *ro*-τ*lacZ*, which labels R2–R5 photoreceptor axons (Garrity *et al.* 1996). To label glia, we immunostained with antibodies against the glial-specific transcription factor Repo (Xiong *et al.* 1994). As expected from our previous X-gal staining analysis (Figure 4), we observed reproducible axon targeting defects upon expression of RNAi against both *sgf11* and *Mp* in glia (Figure 5, C–D, arrowheads). In contrast, glial-specific expression of RNAi against *Luciferase* does not result in any observable axon targeting defect (Figure 5B), and well organized layers of glia are present in the lamina region (Figure 5B, dotted lines). Whereas in *sgf11* mutant larvae, there is a strong glial migration defect (Figure 5A), expression of RNAi against *sgf11* in glia results in a much weaker glial migration phenotype; in contrast to *sgf11* optic lobes in which entire regions of lamina glial cells are missing, expression of RNAi against *sgf11* results in a slight disruption to glial organization (Figure 5C). Surprisingly, this modest defect in glial organization is still associated with mistargeting of a subset of photoreceptor axons (Figure 5C, arrowheads). It is likely that expression of RNAi against *sgf11* does not result in complete loss of Sgf11 protein, however, we cannot confirm this since there is no antibody available to detect Sgf11. Thus, we conclude that knockdown of Sgf11 in glial cells modestly disrupts lamina glial cell migration and/or organization, and correlates with mistargeting of photoreceptor R1–R6 axons.

Similar to expression of RNAi against *sgf11*, expression of RNAi against *Mp* does not cause a significant defect in glial migration. However, we do observe modest disorganization of lamina glia (Figure 5D, arrowheads), and this slight disruption to lamina glial organization is sufficient to result in mistargeting of photoreceptor axons (Figure 5D, arrowheads). These observations show that there is a spatial correlation between defects in glial cell organization and axon mistargeting upon knockdown of *Mp*, indicating that *Mp* is required in glia for proper lamina glial migration and/or organization, which in turn controls photoreceptor axon termination. However, based on these analyses alone, we cannot formally exclude the possibility that *Mp* is also required in glia to provide a termination signal to photoreceptor axons, even though SAGA itself is not.

## Discussion

Here, we identify novel gene targets of the transcription coactivator SAGA in *Drosophila* central nervous system glia. We show that the SAGA-activated gene, *Mp*, is required in glia for proper lamina glial organization and subsequent photoreceptor axon targeting in the optic lobe. Prior to this study, *nonstop*, *sgf11*, and the miRNA *bantam* were the only genes reported to be required in lamina glia for their migration (Li and Padgett 2012).

*Mp* encodes an ortholog of vertebrate collagen XV/XVIII and consists of three major domains: an N-terminal thrombospondin-related domain, triple helix, and a C-terminal Endostatin domain. The identification of *Mp* in this study suggests a novel role for collagen XV/XVIII in glial migration. *Mp* had previously been shown to be required in neurons for correct motor neuron axon pathfinding during embryogenesis (Meyer and Moussian 2009). The C-terminal Endostatin domain of Mp, which can be proteolytically released (Heljasvaara *et al.* 2005), is sufficient for proper motor axon pathfinding in *Drosophila* embryos (Meyer and Moussian 2009). Endostatin, which acts as a signaling molecule (Wickstrom *et al.* 2005), is also necessary for homeostatic synaptic plasticity, and modulates both presynaptic calcium influx and neurotransmitter release (Wang *et al.* 2014). Intriguingly, mutations in human collagen XVIII (*COL18A1*) are associated with Knobloch syndrome, a rare autosomal recessive disorder characterized by severe vision problems including vitreoretinal degeneration (Sertie *et al.* 2000; Bishop *et al.* 2010). In addition, a mutation in *COL18A1* has been identified in an Indian family with SCA7-like symptoms including ataxia and progressive blindness (Paisan-Ruiz *et al.* 2009). Since polyQ-expanded hATXN7 reduces SAGA deubiquitinase activity *in vivo*, in part through sequestration of the ubiquitin protease USP22 (McCullough *et al.* 2012; Yang *et al.* 2015; Lan *et al.* 2015), SAGA deubiquitinase-regulated gene expression is likely to be defective in SCA7 patients. Notably, expression of polyQ-expanded ATXN7 in glia is sufficient to induce neurodegeneration in a SCA7 mouse model (Custer *et al.* 2006), indicating that SAGA deubiquitinase function in glial cells plays a crucial role in SCA7 pathogenesis. This finding is consistent with the general role that glia play in the progression, and in some cases initiation, of neurodegeneration in polyQ-diseases (Lobsiger and Cleveland 2007). Thus, we speculate that downregulation of *COL18A1* expression in glia in SCA7 patients could contribute to the specific visual degeneration associated with this ataxia. However, while defects in expression of other vertebrate collagens (*COL4A1* and *COL4A2*) were identified in microarray analysis of gene expression in a human astrocyte cell culture model of SCA7, *COL18A1* was not identified as being significantly misexpressed (McCullough *et al.* 2012).

An unexpected finding from this study was the overlap between genes that are transcriptionally regulated by SAGA deubiquitinase activity in glia and proteins that are sequestered by polyQ-ATXN7 into inclusion bodies in SCA7. These inclusion bodies that form in the nuclei of neuronal cells result from aggregation of the polyQ-mutant protein, and are a hallmark of polyQ diseases (Holmberg *et al.* 1998; Takahashi *et al.* 2003). Strikingly, several of the major classes of SAGA-regulated genes in glia, including those that regulate protein folding and protein degradation, also form the major protein components of these inclusion bodies. In particular, subunits of the 19S proteasome and the molecular chaperones are both transcriptionally regulated by SAGA in glia (this study) and found in SCA7-associated inclusions (Janer *et al.* 2010; Takahashi *et al.* 2002, 2003; Zander *et al.* 2001; Matilla *et al.* 2001). Notably, one of the most downregulated genes identified in SAGA deubiquitinase mutant glia, *Hsp27*, encodes a molecular chaperone that has reduced levels in SCA7 patients (Tsai *et al.* 2005) and that attenuates polyQ protein toxicity in a *Drosophila* model of neurodegenerative disease when overexpressed (Liao *et al.* 2008). The overlap between transcriptional targets of SAGA in glia and proteins that aggregate in SCA7 inclusion bodies suggests that SCA7 could reduce the levels of proteins involved in protein folding and proteasomal degradation in glial cells both directly and indirectly, through reduced SAGA deubiquitinase activity. It remains to be determined whether this further reduction in the levels of proteins that require SAGA deubiquitinase activity for expression in glia contributes to polyQ-toxicity and neurodegeneration in SCA7.

Previously, we showed that SAGA deubiquitinase activity was important for full expression of tissue-specific genes with developmental functions (Weake *et al.* 2011). When we compare the SAGA deubiquitinase-activated genes in embryonic muscle with those identified in glia in this study, we find that there are only 24 commonly downregulated genes. Since the genes that are activated by SAGA deubiquitinase activity differ so completely in these two different cell types, our findings indicate that the sensitivity of a particular gene to ubH2B-deubiquitination is dependent upon epigenetic factors rather than sequence information such as promoter motifs. Identifying the common chromatin landscape of genes that require SAGA deubiquitinase activity for expression may therefore provide insight into how tissue-specific gene expression is controlled at the chromatin level.

## Supplementary Material

Supplemental Material
