# Supplementary material for: Transcriptome Profiling Identifies Multiplexin as a Target of SAGA Deubiquitinase Activity in Glia Required for Precise Axon Guidance During Drosophila Visual Development
Source: G3 (Bethesda). 2016 Jun 1;6(8):2435–45. doi: 10.1534/g3.116.031310 (PMC4978897; doi:10.1534/g3.116.031310)
Supplement: Supplemental Material [file supp_6_8_2435__index.html]

Transcriptome Profiling Identifies Multiplexin as a Target of SAGA Deubiquitinase Activity in Glia Required for Precise Axon Guidance During Drosophila Visual Development — Supplemental Material 

# Transcriptome Profiling Identifies *Multiplexin* as a Target of SAGA Deubiquitinase Activity in Glia Required for Precise Axon Guidance During *Drosophila* Visual Development

## Supplemental Material for Ma *et al.*, 2016

**Files in this Data Supplement:**

- Figure S1 - qRT-PCR analysis of the GFP transcript level for the four wild-type glial samples used for RNA-seq (post-isolation) compared to each corresponding "pre-isolation" sample. (.ai, 118 KB)
- Table S1 - FPKM values for larval central nervous system (Dm Tissue Expression RNA-seq third instar larvae central nervous system sequences, ModENCODE\_4257: SRR070409, SRR070410) versus glia-enriched nuclear RNA (this study). (.xlsx, 480 KB)
- Table S2 - 966 genes significantly upregulated in nonstop glia compared to wild-type glia based on edgeR analysis with a FDR < 0.01. (.xlsx, 45 KB)
- Table S3 - 836 genes significantly downregulated in *nonstop* glia compared to wild-type glia based on edgeR analysis with a FDR < 0.01. (.xlsx, 39 KB)
- Table S4 - 900 genes significantly upregulated in *sgf11* glia compared to wild-type glia based on edgeR analysis with a FDR < 0.01. (.xlsx, 40 KB)
- Table S5 - 744 genes significantly downregulated in *sgf11* glia compared to wild-type glia based on edgeR analysis with a FDR < 0.01. (.xlsx, 36 KB)
- Table S6 - 779 genes significantly upregulated in both *nonstop* and *sgf11* mutant glia compared to wild-type glia based on edgeR analysis with a FDR < 0.01. (.xlsx, 36 KB)
- Table S7 - 629 genes significantly downregulated in both *nonstop* and *sgf11* mutant glia compared to wild-type glia based on edgeR analysis with a FDR < 0.01. (.xlsx, 34 KB)
- Table S8 - EdgeR output of differential analysis of *nonstop* vs. wild-type glia. (.xlsx, 266 KB)
- Table S9 - EdgeR output of differential analysis of *sgf11* vs. wild-type glia. (.xlsx, 263 KB)
- Table S10 - Primer sequences for qRT-PCR used in this study. (.xlsx, 12 KB)
- Table S11 - Raw counts RNA-seq data for wild-type (wt), *nonstop* and *sgf11* mutant glia. (.xlsx, 1075 KB)
