# Supplementary material for: Transcriptome Profiling Identifies Multiplexin as a Target of SAGA Deubiquitinase Activity in Glia Required for Precise Axon Guidance During Drosophila Visual Development
Source: G3 (Bethesda). 2016 Jun 1;6(8):2435–45. doi: 10.1534/g3.116.031310 (PMC4978897; doi:10.1534/g3.116.031310)
Supplement: Supplemental Material [file supp_g3.116.031310_FigureS1.ps]

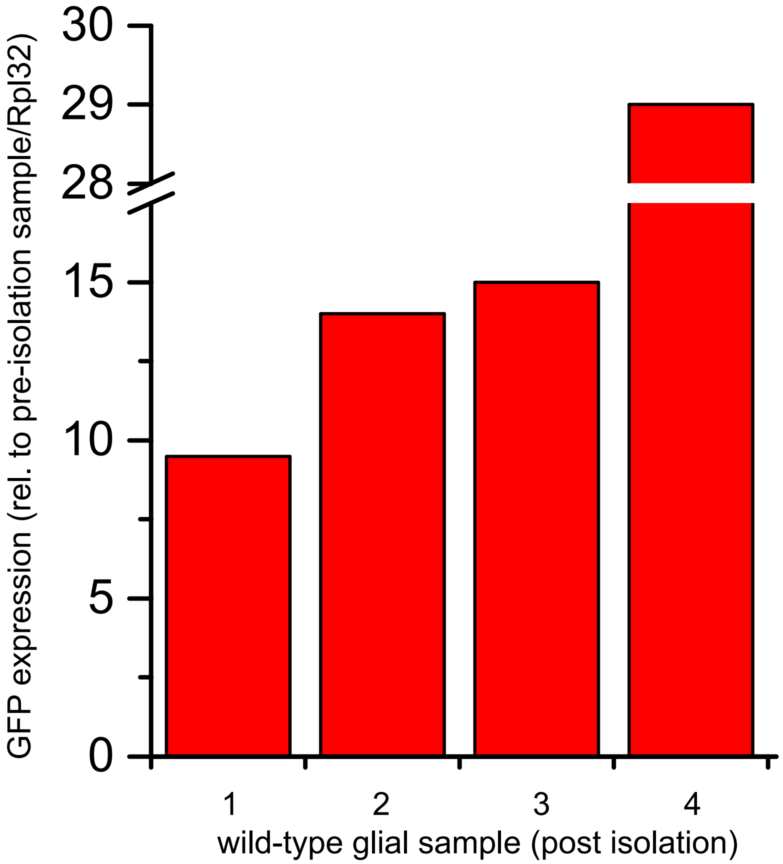

Supplemental Figure 1. qRT-PCR analysis of the GFP transcript level for the four wild-type glial samples used for RNA-seq (post-isolation) compared to each corresponding "pre-isolation" sample. For each post-isolation sample, GFP transcript level is normalized to RpL32 transcript level and is plotted relative to the pre-isolation sample, which is set to one.
